# Rearing enhancement of *Ovalipes trimaculatus* (Crustacea: Portunidae) zoea I by feeding on *Artemia persimilis* nauplii enriched with alternative microalgal diets

**DOI:** 10.1038/s41598-020-67933-3

**Published:** 2020-07-02

**Authors:** Antonela Martelli, Elena S. Barbieri, Jimena B. Dima, Pedro J. Barón

**Affiliations:** 1 Laboratorio de Oceanografía Biológica (LOBio), Centro para el Estudio de Sistemas Marinos – CONICET, Complejo CCT CONICET-CENPAT, Blvd. Brown 2915, (9120) Puerto Madryn, Chubut, Argentina; 2 Laboratorio de Cefalópodos, Instituto de Biología de Organismos Marinos – CONICET, Complejo CCT CONICET-CENPAT, Blvd. Brown 2915, (9120) Puerto Madryn, Chubut, Argentina

**Keywords:** Biological techniques, Developmental biology

## Abstract

The southern surf crab *Ovalipes trimaculatus* (de Haan, 1833) presents a high potential for aquaculture. In this study, we analyze the benefits of different dietary treatments on its molt success and fitness of larval stages. *Artemia persimilis* nauplii were enriched with monospecific (*Nannochloropsis oculata, Tetraselmis suecica, Dunaliella salina, Isochrysis galbana and Chaetoceros gracilis*) and multispecific (Mix) microalgal diets twice a day over a 48-h period. Mean total length (TL), growth instar number (I) and gut fullness rate (GFR) of nauplii showed significant differences between dietary treatments at several sampling times, optimal results being observed in those providing Mix. *Artemia* nauplii grown under most experimental dietary treatments reached the capture size limit for *Ovalipes trimaculatus* zoea I (700 µm) within 24 h. After that time interval, Mix-enriched nauplii were amongst those with higher protein contents. *Ovalipes trimaculatus* zoea I fed on *Artemia* nauplii enriched during 24 h under different dietary treatments showed significant differences in survival, inter-molt duration, molting success to zoea II and motility. Optimal results were observed in zoea I fed on Mix-enriched *Artemia* nauplii. This work not only represents a first step towards the dietary optimization for *O. trimaculatus* zoeae rearing but also provides the first results on the use of enriched *A. persimilis*.

## Introduction

Portunid crabs stand out as highly valued resources for fisheries and aquaculture because of their export potential and high nutritional value^1^. Due to their size, meat content and unique flavor, their products are highly priced in domestic and international markets^2^. Since global portunid product demands exceed expectations each year, world fisheries captures have grown steadily, surpassing 1 million tons by 2016^3^, leading to the local overexploitation of some species^4^. At the same time, unsatisfied market demands have been increasingly sustained by restocking and aquaculture production^5^ in excess of 0.38 million tons by 2016, 96% of which were produced in East Asia^3^.

At present, portunid aquaculture is restricted to meat production of *Scylla serrata*, *Portunus pelagicus*, *Portunus trituberculatus*, *Portunus sanguinolentus* and *Charybdis feriata*^1^ and to restocking of *Callinectes sapidus*^6^. Still, the culture potential for many other large-sized species from the taxon is virtually unexplored. Indeed, reported production out of the west coast of Asia is still negligible^3^, representing both a challenge and an opportunity for the industry sector elsewhere. The southern surf crab *Ovalipes trimaculatus* (de Haan, 1833), one of the species with high potential for aquaculture, is widely distributed in coastal areas of the South Atlantic, Indian and Pacific Oceans, being present along the mid-latitude (25°–45° S) Argentinean coast^7^, where populations have been targeted by artisanal fisheries over the last 10 years providing products with good acceptance in the local shellfish markets^8,9^. Although several studies have been conducted on the structure of its populations, reproduction, growth and some behavioral and anatomical aspects^10–15^ information available on the biology of its early life stages is still scarce and insufficient to allow encouraging their breeding in aquaculture facilities^12,16,17^.

Larval stages of decapod crustaceans may be lecithotrophic or planktotrophic depending on the reproductive strategy of each species^18^. While the former cover their food requirements by consuming abundant yolk reserves stored in the oocytes, the later start feeding on different plankton components soon after hatching^18,19^. Thus, breeding planktotrophic decapod larvae requires assessing the quality and frequency of food consumption to optimize survival, growth and physiological condition^20^. However, this involves the simultaneous maintenance of larvae and auxiliary food cultures, representing the bottleneck for the aquaculture of many decapods, including portunid crabs^21,22^.

Research on dietary quantity and quality requirements has contributed to minimize mortality and to enhance growth and fitness of zoeae from several portunids including *Scylla serrata*^23^, *Portunus sanguinolentus*^24^, *P. pelagicus*^25^ and *Callinectes sapidus*^26^. Among other live feeds, brine shrimps (*Artemia *spp.) are widely used due to their food carrying capacity and good acceptance^27–29^. At low temperatures (i.e., 12°), like those experienced by *O. trimaculatus* during the reproductive season, *Artemia persimilis*, a brine shrimp species native from Argentina and Chile^30^, shows higher survival rates compared to its native congener *A. franciscana*, probably resulting from its adaptation to Patagonian climate conditions^31,32^. Its cysts have nutritional properties comparable to those of other commercial species traded in international markets^33^^,^ displaying high hatching efficiencies^34^ and producing small-sized nauplii with elevated fatty acid unsaturation, highly desirable for use as live food in aquaculture^35,36^. Still, up to date the species has been rarely used to feed larval stages of fishes or marine invertebrates^37^.

*Artemia* is an incomplete food source itself because of the paucity of some essential elements in its composition, as for example the n3 and n6 polyunsaturated fatty acids (PUFAs) frequently required for successful development of crustacean larvae^28^. Although nutritive commercial emulsions have been used to complement its composition, fulfilling the dietary requirements of larvae preying on it, their autoxidation with synthesis of toxic compounds^38^ along with relatively high commercial cost^39^ have discouraged this practice. Alternatively, feeding *Artemia* with various types of food in suspension culture systems has allowed its enrichment with higher fatty acid content and to use it as carrier of other nutrients (e.g., vitamins), antimicrobial substances, vaccines and probiotics^40^.

Microalgae can be incorporated as a food additive to supply basic nutrients into a wide variety of food, and represent an alternative to replace feedstuff and ensure sustainability standards in aquaculture. Their positive effect on the growth rate and physiological condition of aquatic species are related to their increased triglyceride and protein deposition in muscle, improved resistance to diseases, decreased nitrogen output into the environment, and augmented omega-3 fatty acid content, physiological activity and carcass quality^38,41^. Typically, microalgae can provide up to 30–40% protein, 10–20% lipid and 5–15% carbohydrate contains if feed to *Artemia* during the exponential phase of culture growth^41^^,^ representing an energy source with a high benefit to cost ratio^38^. Thus, finding an optimal dietary combination of microalgae and an appropriate schedule for feeding them to *Artemia* are critical to guarantee their nutritional value at low production cost^38,42^.

Taking into consideration all of the above mentioned, this study has two main objectives: (1) testing alternative microalgae dietary compositions and different feeding schedules to enrich *Artemia persimilis* so as to optimize its nutritional value as live food, and (2) enhancing survival, growth and physiological condition *of Ovalipes trimaculatus* zoeae I by feeding them with *Artemia persimilis* nauplii enriched on different microalgal diets.

## Materials and methods

### Effects of different microalgae dietary treatments on the condition of *Artemia* nauplii

Five species of microalgae: *Nannochloropsis oculata*, *Tetraselmis suecica*, *Dunaliella salina*, *Isochrysis galbana*, *Chaetoceros gracilis* were cultured separately to feed *Artemia* in monospecific diet treatments, and also all of them were combined in equal proportions to constitute a multispecific diet treatment (Mix) Table 1. Microalgae were cultured in autoclaved 0.45 um-filtered and UV-treated seawater using f/2 nutrient medium at 30 psu^43^. In the case of *Chaetoceros gracilis*, the culture medium was supplemented with a silicate solution (30 mg L^−1^). Microalgae cultures were supplied with constant aeration and were subjected to a 12:12 h light:dark photoperiod and 22° ± 1 °C temperature in a culture chamber. Daily, number of cells per ml were determined using a Neubauer chamber and cultures were maintained at 10^6^ cell mL^−1^. Once cell populations reached the exponential growth phase (i.e., 5 days from inoculation), the culture was used to feed *Artemia*. The ratio of microalgae to *Artemia* was kept constant throughout the experimentation period^44^.

**Table 1:** Dietary treatments applied for enrichment of *Artemia nauplii*.

Treatment	Short name
Equal proportions of the five microalgal species	Mix
*Dunaliella salina*	Duna
*Nannochloropsis oculata*	Nanno
*Isochrysis galbana*	Iso
*Tetraselmis suecica*	Tetra
*Chaeroceros gracilis*	Chaeto

*Artemia* cysts (Biosima, *A. persimilis*) were disinfected with a sodium hypochlorite solution (5 mg L^−1^ of active chlorine) during 10 min. Then, they were washed with freshwater to remove any rest of disinfecting solution. Cleaned cysts were incubated in sterile diluted seawater at 35 °C and 15 g L^−1^ of salinity under continuous aeration and 2000-lx illumination until hatching^45^. Twenty four hours later, newly hatched nauplii (as maximum 24 h alive) were collected using a 250-µm mesh-size net and transferred into cylindroconical recipients containing 1 L of sterile seawater with constant aeration. All of the experiments were conducted in an incubator chamber under controlled temperature (26 °C) and photoperiod (12:12 h light:dark).

To test the six feeding treatments Table 1, initial density of recently hatched (< 24 h) *Artemia* nauplii in the containers was adjusted to approximately 400 nauplii L^−1^. Microalgae cultures were kept at nearly 10^6^ cells mL^−1^ throughout the experiment to maintain constant cell biochemical composition^44^. Throughout the 48 h experiment duration, *Artemia* nauplii were fed twice: at the beginning of the experiment and after 24 h, with 10^6^ cell mL of microalgae. Samples for growth, gut fullness, and biochemical determinations were taken as described in Table 2, using a hand net 150 μm in mesh size. The sampling interval was established according to growth and molt stages of nauplii at 26 °C reported by Cohen et al.^46^ and considering the results of previous observations on the time required by *Artemia* to achieve gut fullness.

**Table 2:** Sampling and feeding schedule applied on *Artemia nauplii*.

Time (h:min)	00:00	00:15	00:30	12:00	12:15	12:30	24:00	24:15	24:30	36:00	48:00
Feeding	x						x				
Sampling	x	X	x	x	x	x	x	x	x	x	x

Samples of *Artemia* nauplii were taken by duplicate every 12 h since the beginning of the experiment to evaluate the proximal composition under each enriching treatment. Gross biochemical composition of *Artemia* nauplii was based on classical methods^47^. Moisture content was determined by drying the sample in an oven at 105 °C to constant weight^48^. Ash content was obtained by drying the samples in a furnace at 550 °C for 8 h^48^. Two key nutritional components were determined: (i) fat content was established by using the Bligh Dyer extraction method^49^^,^ and (ii) crude protein content was estimated by a colorimetric method^50^. All results were expressed as mg g^−1^ of proximal composition in dry weight.

To determine growth and gut fullness, 10 *Artemia* nauplii were sampled for measurements at each sampling time for each dietary treatment. Individuals were handled with forceps under a Leica (DM2500 model) dissecting stereomicroscope at 50X magnification. Total length (TL), total gut length (Tgl) and total full gut length (Tfg) were measured with the Leica Application Swite program (V 4.5). Based on these registers, the rate of gut fullness (GFR) was calculated for each individual as:

$$\mathrm{G}\mathrm{F}\mathrm{R}=\frac{Tfg}{Tgl} \times 100$$

This ratio was used as an indicator of the carrying capacity of nutritional components into the guts of the *Artemia* nauplii. Growth stages were determined in samples preserved in 70% ethanol solution, based on total lengths and on the presence of specific appendages, following Cohen et al.^46^.

### Experiment II: effects of different dietary treatments on the condition of *Ovalipes trimaculatus* zoeae I

Ovigerous *O. trimaculatus* females carrying embryos at advanced stages of development (Stages IV and V^16^) were hand-collected by SCUBA diving on subtidal sand bottoms of Nuevo Gulf (42° 25′ S, 64° 07′ W, Argentina) during the reproductive season (October–December) of 2015. Specimens were transported to the Experimental Marine Aquarium of the National Patagonian Sci-Tech Center (CCT CONICET-CENPAT) and were acclimated in plastic tanks containing filtered seawater with continuous aeration. Up to 30% of the seawater volume was renewed once a day until hatching occurred. After hatching, groups of 100 zoeae I were collected using a glass pipette and placed into 2 L containers filled with sterile seawater under 13 ± 1 °C and 33 ± 1 g L^−1^, simulating the average of SST and salinity conditions through the reproductive season in Nuevo Gulf^51^. Photoperiod was fixed to 12:12 h, simulating the natural light cycle at the time of experimentation.

Newly hatched *O. trimaculatus* zoeae I were subjected to nine dietary treatments Table 3, each applied on 3 replicates of the 100-zoeae I samples mentioned above. Except for the starving treatment, zoeae were fed with 2–4 *Artemia* nauplii mL^−1^ complemented with Mix at constant microalgae to *Artemia* ratio (10^6^ cells mL^−1^)^44^. Whenever corresponded, *Artemia* nauplii were enriched during 24 h before being offered to the zoeae, taking into consideration the results of Experiment I. Every morning the zoeae were transferred into a new beaker using a 5 mL pipette, counted and staged based on their total length, presence of appendages and shape of the eyes, following the descriptions of Schoeman and Cockcroft^17^. For each treatment, duration from hatching to the first moult (D, in days), survival (S) and the number of zoeae II (nZII) obtained from the total number of zoeae I at the beginning of the experiment, were registered. Moulting success (MS %) was calculated as the number of live zoeae II over nZII.

**Table 3:** Dietary treatments applied to *Ovalipes trimaculatus* zoeae I.

Treatment	Treatment short name
1. *Artemia* enriched with Mix microalgal	A Mix
2. *Artemia* enriched with *Dunaliella salina*	A Duna
3. *Artemia* enriched with *Nannochloropsis oculata*	A Nanno
4. *Artemia* enriched with *Isochrysis galbana*	A Iso
5.*Artemia* enriched with *Tetraselmis suecica*	A Tetra
6. *Artemia* enriched with *Chaetoceros gracilis*	A Chaeto
7. Starved *Artemia*	A sta
8. Only Mix of microalgae	Mix
9. Starved Zoea	Z sta

Motility was determined as a measure of larval fitness based on the vertical displacements (Vd, cm seg^−1^) of zoeae I in response to the light stimulus. After feeding for 5 days, 10 zoeae I were randomly chosen from each treatment, and placed in a rectangular glass column (15 cm high × 10 cm long × 1 wide) previously filled with 150 mL of sterilized seawater conditioned at the same temperature as the larval culture. The column was placed within a dark box equipped with a white 2000-lm led-light set on the top. Taking into account their observed positive phototaxism and high photokinesis, each *O. trimaculatus* zoea I was left at the bottom of the column and light was immediately turned on. Time (in seconds) taken by the zoeae to displace from the bottom to the top of the column was registered with a digital chronometer, and vertical displacement (Vd) was calculated thereafter. In a preliminary pilot experiment testing the time taken by 150 zoea I to displace from bottom to top of the column, most passed the 10-cm mark in an average of 32.97 s, and it took more than 100 s only to three of them (maximum time = 112.01 s). For this reason, while testing for different treatments, whenever a zoea did not reach the top after 3 min the experiment was stopped.

### Data analysis

One-way analysis of variance (ANOVA) was used to test mean differences between treatments for each variable of Experiment I whenever normality (Kolmogorov–Smirnov test) and homoscedasticity (Fisher test) assumptions were fulfilled. Square root transformation was applied if necessary. When normality or homoscedasticity assumptions could not be confirmed, the nonparametric Kruskal–Wallis test was used to examine differences between treatments, especially in Experiment II. Whenever differences were significant (P < 0.05), Tukey and Dunn post hoc tests were used for treatment comparisons.

## Results

### Experiment I: effects of different microalgae dietary treatments on the condition of *Artemia* nauplii

**Figure 1:**
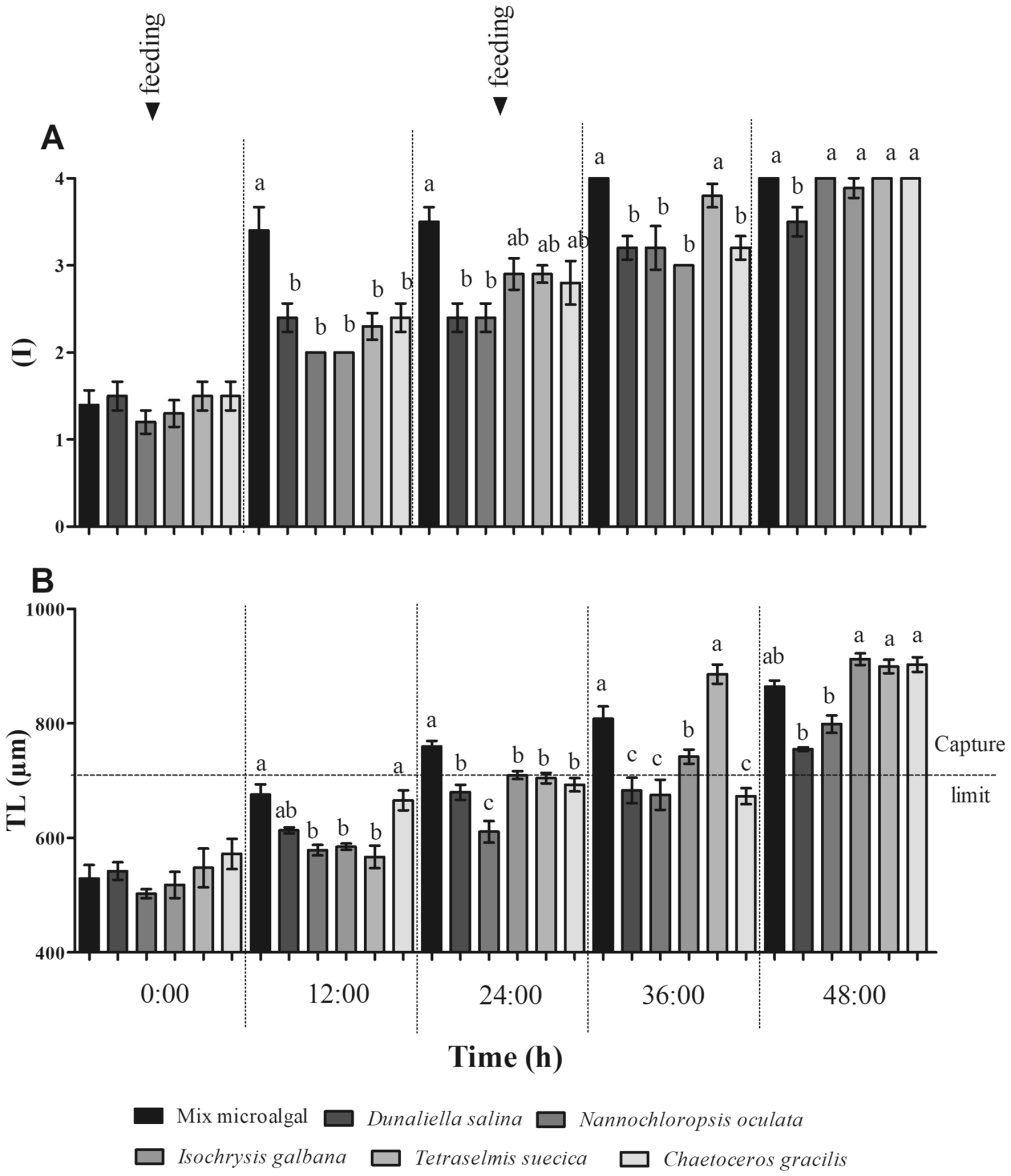
Development and growth of *Artemia* nauplii under different dietary treatments: (**A**) average naupliar instar (I), and (**B**) total length (TL), at different times after hatching. Bar heights and whisker amplitudes represent mean ± standard deviation values respectively. Different letters on top of the bars indicate significant differences revealed by Tukey tests.

Dietary treatments applied on *Artemia* nauplii resulted in significant differences in mean TL (µm) and growth of instar (I) at every sampling time starting on 12 h Table 4. On the average, nauplii fed on Mix grew to instar 3 after only 12 h, significantly faster than those fed on monospecific microalgal cultures Fig. 1A. Also, larvae fed on Mix displayed one of the highest mean TL at all sampling times, while those supplemented with Nanno performed one of the lowest Fig. 1B. Since estimated mean (± sd) length reported for *O. trimaculatus* zoeae I was 1.9 ± 0.19 mm^52^, preys larger than 700 µm were beyond their capture limit. Therefore, a feeding period not longer than 24 h resulted optimal for most dietary treatments Fig. 1B.

**Table 4:** ANOVA tests for differences in means of total length (TL), instar (I) and gut fullness rate (GFR %) between dietary treatments applied for enrichment of *Artemia* nauplii at different times in Experiment I.

Time (h)	SC	d.f	CM	F	P-value
**Total length (µm) (TL)**					
0	29,732.27	5	5,946.45	1.12	0.3607
12	79,767.86	5	15,953.57	9.29	**< 0.0001**
24	79,153.98	5	15,830.8	15.3	**< 0.0001**
36	329,571.09	5	6,314.22	19.19	**< 0.0001**
48	159,656.2	5	31,931.24	34.63	**< 0.0001**
**Growth instar (I)**					
0	1.35	5	0.27	1.08	0.3819
12	13.28	5	2.66	10.79	**< 0.0001**
24	6.95	5	1.39	4.49	**0.0017**
36	8	5	1.6	8.31	**< 0.0001**
48	2	5	0.4	6.26	**0.0001**

Time (h:min)	Gut fullness rate (GFR)				
00:15	424.75	5	84.95	27.3	**< 0.0001**
00:30	5.65	5	1.13	3.58	**0.0075**
12:00	539.94	5	107.99	23.85	**< 0.0001**
24:00	535.76	5	107.95	35.49	**< 0.0001**
24:15	5,873.49	5	1,174.70	8.18	**< 0.0001**
24:30	1.77	5	0.35	1.01	0.4189
36:00	197.91	5	39.58	5.67	**0.0003**
48:00	177.49	5	35.5	3.56	**0.0076**

P values in bold letters denote significant statistical differences.

**Figure 2:**
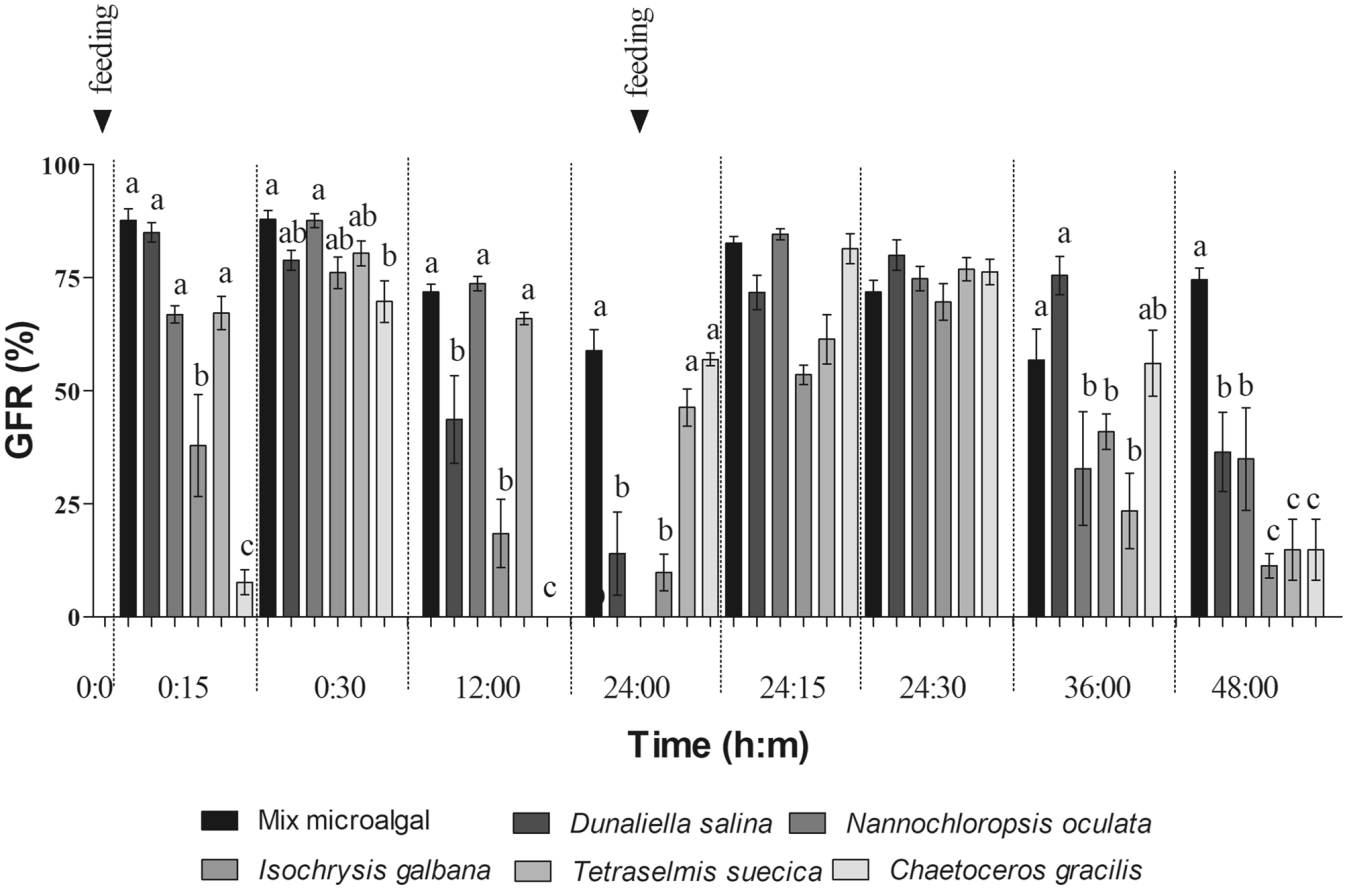
Gut fullness rate (GFR%) of *Artemia* nauplii at different times and dietary (enrichment) treatments. Bar heights and whisker amplitudes represent mean ± standard deviation values respectively. Different letters on top of the bars indicate significant differences revealed by Tukey tests.

With the exception of *Artemia* nauplii enriched with Chaeto and Iso, nauplii showed a rapid ingestion of microalgae with high mean gut fullness (%) just 15 min after feeding Fig. 2. At all sampling times except for 24 h 30 min, gut fullness (%) showed significant differences between feeding treatments. In general, *Artemia* nauplii fed on Mix were among those with the highest gut fullness (%), while those fed on Chaeto, Iso and Dunna showed a fast gut emptying Fig. 2.

Just after hatching, *Artemia* nauplii showed relatively low contents of all measured biochemical components Table 5. Proximal composition was not measured right after first food supply, considering that instar I nauplii do not feed, however, it was observed that protein and lipid representation relative to total dry matter peaked 30 min after the second feeding for all dietary treatments, reflecting the enhancement provided by microalgae encapsulation Table 5. At that time, all treatments except Tetra provided high percentages of lipids, with Iso presenting the highest value. Mix and Iso supplied the highest percentages of protein Table 5.

**Table 5:** Biochemical composition of *Artemia nauplii* enriched under different dietary treatments in Experiment I, expressed as mean mg/g of dry matter.

mg g^−1^	Time (h min)	Mix	Duna	Nanno	Iso	Tetra	Chaeto
Ash	12	77.2	62.7	104.1	57.8	77.1	71.4
	24.3	69.3	34.7	105.8	69.4	80.9	77.8
	36	75.5	63.2	122.4	68.4	84.5	88.5
	48	89.9	61	107.1	75.3	109.4	92.6
Protein	12	426	389.1	445	355.5	237.8	418
	24.3	774.4	632.5	449.2	797.5	493.4	626.1
	36	472.3	436.8	583.4	474	778.3	741.4
	48	515.7	592.5	470.3	669.4	663	731.8
Lipids	12	35.4	51.6	24.4	50.6	68.7	44.6
	24.3	36.9	49.2	30.5	77.9	45.2	46.2
	36	26.4	26.6	28.1	34.5	105.7	40.4
	48	27.2	49.4	30.2	51.3	68	116.4

Please see short name in Table 1.

### Experiment II: effects of different dietary treatments on the condition of *Ovalipes trimaculatus* zoeae I

**Figure 3:**
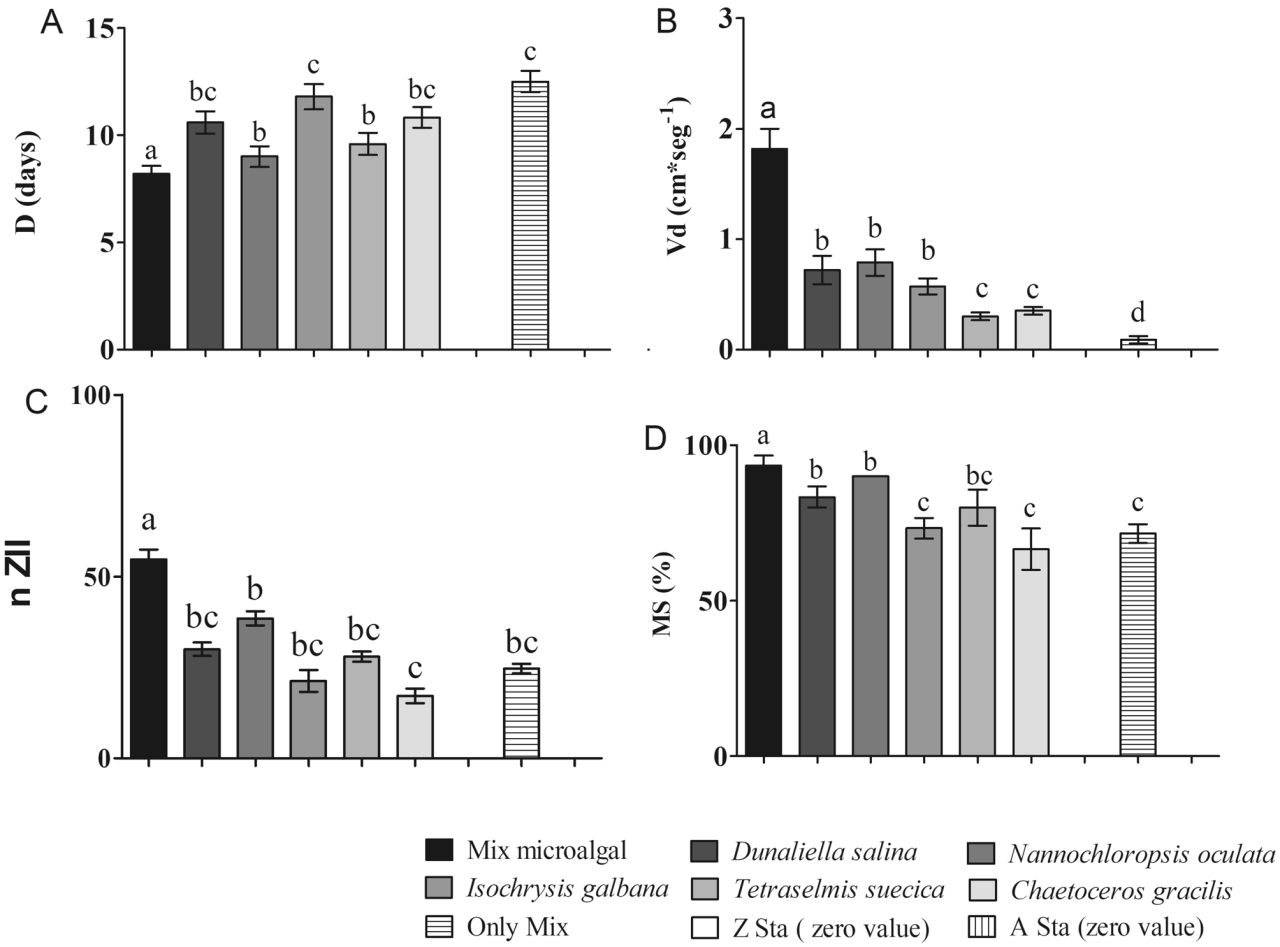
Growth, survival and physiological condition of *Ovalipes trimaculatus* zoeae I on different dietary treatments. (**A**) Zoeae I intermolt duration (**D**); (**B**) Vertical displacement average speeds (cm s^−1^) (Vd); (**C**) Total number of live and dead zoeae II obtained in each treatment (nZII); (D) Molting success to zoeae II as the ratio of the number of life zoeae II over nZII (MS %). Bar heights and whisker amplitudes represent mean ± standard deviation values respectively. Different letters on top of the bars indicate significant differences revealed by Dunn tests.

Different dietary treatments applied to *O. trimaculatus* zoeae I resulted in significant differences in survival, intermolt duration, vertical displacement and molting success Table 6. Zoeae I fed on Mix-enriched *Artemia* nauplii presented the shortest intermolt duration, while those fed on unencapsulated Mix or on nauplii enriched with Dunna, Iso or Chaeto presented the longest Fig. 3. *Z*oeae I fed on unenriched *Artemia* nauplii, and those that were starved, survived 6 days and did not molt Fig. 3. Furthermore, Zoeae I from treatments where they were starved or feed on starved *Artemia* (Z sta and A sta, Table 3 were the only that could not displace to the top of the glass column within the stipulated time (180 s). Feeding *O. trimaculatus* zoeae I on Mix-enriched *Artemia* nauplii resulted in the highest mean survival and molting success (%) Fig. 3. In contrast, feeding with unencapsulated Mix, or *Artemia* nauplii enriched with Iso or Chaeto resulted in relatively low values for this variable Fig. 3. In agreement with previous results, Zoeae I fed on Mix-enriched *Artemia* nauplii also showed the highest vertical displacement, while those fed on unencapsulated Mix displayed the lowest values for this variable Fig. 3.

**Table 6:** Kruscal–Wallis tests for differences in survival (S (%)), zoeae I intermolt duration (D), vertical displacement (Vd), total number (live and dead) of zoeae II (*nZII*) and molting success (MS (%)) between groups *Ovalipes trimaculatus* zoeae I fed on different diets.

Variable	Kruskal–Wallis test	
	H	P-value
S (%)	67.23	**0.0489**
D (days)	41.26	**0.0001**
Dv (cm seg^−1^)	50.17	**0.0001**
nZII	33.62	**0.0001**
MS (%)	91.22	**0.0001**

P values in bold letters denote significant statistical differences.

## Discussion

Several studies have revealed the effects of different diets on growth, development, ingestion capacity and biochemical composition of enriched *Artemia* nauplii and of marine invertebrate and fish larvae feeding upon them^29,53^. Although enrichment diets include a wide variety of inert and live foods, the use of microalgae as a food with well-known nutritional properties is one of the most frequent choices. Indeed, they could represent the best food formula at the lowest cost^54^. Microalgae selected for this study have been characterized by their high contents of aminoacids (e.g., Chaeto and Nanno), lipids (e.g., Iso) and PUFA (e.g., Nanno, DHA-rich Iso, C20-rich Tetra), and carotenes (e.g., Dunna and asthaxanthin/lutein-rich Tetra)^45,55^. All of them provided *Artemia* nauplii the nutritional complements necessary for their growth to the capture size limit for *O. trimaculatus* zoeae I (i.e., 700 µm), and all except Duna and Nanno did it within the first 24 h probably because of their low digestibility^29^. Moreover, nauplii fed on a mix of identical proportions of the five microalgal species displayed an optimum growth, nutrient carrying capacity and biochemical composition soon after enrichment, in agreement with previous studies^56^. *Artemia* nauplii enriched on Chaeto did not grow faster than those fed on Iso*,* both after 24 or 48 h, in agreement with results reported by Lora-Vilchis et al.^57^.

The quantity of microalgae remaining in the digestive system of enriched *Artemia* nauplii during a certain period of time, reflected by the gut fullness in this work, is relevant to evaluate the effectiveness of the enrichment treatment. On one hand, starvation occurring just after enrichment of *Artemia* nauplii results in a progressive decline of gut and tissue nutrient reserves and on a change of their nutritional value over the time^58^. Since larvae from some crustacean decapods, such as *Jasus edwarsii*, tear enriched *Artemia* into pieces previous to their ingestion, causing the loss of its microalgal gut contents, attention has been focused on their relative contribution to the overall biochemical composition of nauplii and juveniles of the species^59^. Based on that study, and considering that *Artemia* nauplii are non-selective filter-feeders with a relatively high ratio of gut content to body volume^60^, it can be assumed that microalgae in gut contents of enriched specimens represent their main nutrient reservoir, and therefore highest gut retention of Mix observed in this study suggest that this is the optimum dietary treatment. Since high gut fullness observed in Nanno and Tetra 12 h after first feeding could not be observed 12 h after the second feeding (36 h), it is difficult to draw clear conclusions on these treatments. Lowest gut fullness observed in Dunna*,* Iso and Chaeto reflected fast emptying in the starving period, and probably a low efficacy of enrichment if nauplii are offered as food few hours after enrichment. Finally, since gut fullness of *Artemia* nauplii was high for all treatments during 15–30 min after enrichment, it is recommended for an effective incorporation of microalgae not to exceed this time period when offering *Artemia* nauplii to the zoeae.

The nutritional value of microalgae can vary significantly depending on the culture conditions^61^. Therefore, a careful selection of a mixture of microalgae harvested at the time of optimum physiological condition (exponential growth phase^62^, should provide an excellent nutritional package for larval stages of many marine invertebrates. Dhont and Van Stappen^60^ inform that for a variety of *Artemia* strains, biochemical composition of nauplii range within 416–619 mg g^−1^ for protein, 120–272 mg g^−1^ for lipids and 71–214 mg g^−1^ of ash. In this study, protein contents were equal to or higher than values reported by these authors, particularly for those obtained 30 min after the second feeding. In contrast, the representation of lipids in *Artemia* nauplii total dry weight was relatively low compared to values reported by Dhont and Van Stappen^60^, ranging from 40 to 80 mg g^−1^ 30 min after the second feeding, and exceeding this range only in Tetra (≈100 mg g^−1^ after 36 h) and Chaeto (≈ 120 mg g^-1^ after 48 h).

Although no information is yet available on the specific nutrient requirements of *O. trimaculatus* zoeae I, and little is known about those of the broad diversity of brachyuran species^63^, there is some evidence pointing out that 300 mg g^−1^ of protein in dry matter of microalgae selected for aquaculture satisfy nutrient demands of zoeae from crustacean decapods^60^. In fact, most microbound or microencapsulated diets formulated for crustacean larvae contain between 300 and 500 mg g^−1^ of crude protein to cover their nutrient demands (Holme et al. 2006). Therefore, protein content of *Artemia* nauplii in excess of 300 mg g^-1^ of dry matter registered in this study 30 min after the second feeding event (i.e., at time 12 h 30 min) should cover the nutritional requirements of *O. trimaculatus* zoea I. On the other hand, lipid requirement of crustacean larvae of many cultured species range within 43–130 mg g^−1^ (Holme et al. 2006). Although values registered in enriched *Artemia* nauplii in this study should satisfy the lipid requirements of larvae of many of these species, lipid contents resulted low compared to those of many *Artemia* strains^60^.

Since the early work of Benijts et al.^64^, demonstrating that dry weight as well as caloric, lipid and fatty acid contents decrease as *Artemia* pass through successive molts, there is agreement in that enriched *Artemia* must not be reared for more than 24 or 36 h until being offered as live food in aquaculture production. Indeed, Sorgeloos et al.^28^ recommend not exceeding a 24-h incubation period after hatching. On the other hand, since the average length (± sd) of *O. trimaculatus* zoeae I, measured from the tip of the spine to the telson, is 1.9 mm (± 0.19 mm)^52^, offering large *Artemia* nauplii is expected to impose anatomical constraints for their capture and manipulation. Although this study does not provide a strict evaluation of the optimum size relationship between *Artemia* nauplii and *O. trimaculatus* zoeae I, direct observations made during the experiments showed that the portunid larvae are not able to capture *Artemia* larger than 700 μm^52^. On the other hand, even if the zoeae of *O. trimaculatus* were capable to fed on nauplii larger than that size by tearing apart their body parts, as observed for the zoeae of *Scylla serrata*^65^^,^ this could result in the loss of nutritional reserves in the gut contents of brine shrimps, as pointed by Smith et al.^59^, suggesting that this practice should be avoided.

Considering that newly hatched zoeae I of portunids have low motility capacity compared to later larval stages, it has been argued that they catch food items mainly by chance and consequently feed less frequently (^66^, Holme et al. 2006). Concentrations of 2–4 *Artemia* nauplii ml^-1^ provided to *O. trimaculatus* zoeae I in this study have been reported to successfully sustain the survival and growth of *Scylla tranquebarica* from zoea I to megalopa^25^. However, the later and other studies have stated that survival can be enhanced when *Artemia* nauplii are complemented with rotifers^67^. Furthermore, rotifers have been used as the only source to feed *Callinectes sapidus* zoeae I and II, supplementing with *Artemia* nauplii from zoeae III to VIII^68^. Nevertheless, zoeae I of *C. sapidus* present markedly smaller total lengths (0.90–1.25 mm)^69^, than those of *O. trimaculatus*. In this study, highest survival, molting success and vertical displacement, and lower intermolt duration were obtained when feeding was based on MIX-enriched *Artemia*. In contrast, zoeae I fed on unenriched nauplii survived until the sixth day and could not molt to the next instar, confirming that it offers a reduced nutritional contribution^29,70^. On the other hand, provision of *Artemia* nauplii enriched with monocultures of different species of microalgae resulted in intermediate growth, survival, development and physiological conditions of *O. trimaculatus* zoea I. All of these results, however, should be considered with care since effects of dietary treatments applied to *O. trimaculatus* zoeae I could express in later developmental stages, as observed on *Portunus trituberculatus* zoeae fed on Nanno-enriched *Artemia*, where despite high zoeal growth and survival, the first postlaval instar (i.e., crab I) presented anomalous morphology and were inviable^29^.

Crab zoeae are relatively strong swimmers, having the capacity to respond to a variety of external stimuli including light, gravity, salinity and temperature, among others^71^. Zoeae I of *O. trimaculatus*, as those of other portunid crabs^72^, present a strong positive phototaxis. However, stressors such as elevated concentration of CO_2_^73^ or other organic and inorganic chemicals^74^ in seawater, and low physiological condition associated to starvation or sub-optimal feeding^20^, may affect the phototactic response and swimming capacity of their larvae as we can observed in the starvation treatment of Experiment II. Therefore, differences in vertical displacement records of *O. trimaculatus* zoeae are likely the result of contrasting phototactic and photokinetic responses associated to the physiological condition of zoeae fed on different diets, presumably optimal for the Mix treatment.

This work represents a step ahead towards the dietary optimization for rearing of *O. trimaculatus* zoeae, that, along with other recent studies^14,16^, is expected to contribute building the necessary knowledge to help producers reducing time and cost associtated to the species’ larval breeding process^54,75^.
